# When Do Narcissists Burn Out? The Bright and Dark Side of Narcissism in Surgeons

**DOI:** 10.3390/ijerph192215123

**Published:** 2022-11-16

**Authors:** Madiha Rana, Erik Riedel, Franziska Czens, Hendric Petersohn, Henriette L. Moellmann, Lara Schorn, Majeed Rana

**Affiliations:** 1 Department of Psychology, European University of Applied Sciences for Distance Learning Hamburg, Doberaner Weg 20, 22143 Hamburg, Germany; 2 Department of Oral, Maxillo- and Plastic Facial Surgery, Heinrich-Heine-University, Moorenstr. 5, 40225 Duesseldorf, Germany

**Keywords:** narcissism, admiration, rivalry, burnout

## Abstract

This study addresses narcissism as an important psychological factor for the prediction of burnout. Previous research has produced inconsistent findings on whether narcissism is beneficial or detrimental to the development of burnout which is due to the fact that narcissism is viewed as an overall construct rather than on a dimensional level. This study applied a two-dimensional approach to narcissism in burnout. Three hundred-fifty-two surgeons from Germany were asked to complete the Maslach Burnout Inventory and the Narcissistic Admiration and Rivalry Questionnaire. Linear regression analyses revealed that high scores in admiration predicted high personal fulfillment, low emotional exhaustion, and depersonalization. For rivalry, the opposite picture emerged. The results indicate that admiration seems to have a protecting effect, whereas rivalry appears to promote burnout severity.

## 1. Introduction

### 1.1. Burnout in Surgeons

Burnout syndrome describes an individual response to chronic work stress that develops gradually and eventually becomes chronic including possible health changes [1]. Psychological damage occurs on a cognitive, emotional, and attitudinal level, manifesting itself in negative behavior for example towards work or colleagues [2]. Freudenberger et al. introduced the term “burnout” in the early 1970s into the social field [3] and described it as a state of exhaustion, fatigue, and frustration due to a professional activity not fulfilling aniticipated expectations. The term was further developed by Maslach et al. [4] who defined burnout as a gradual process of emotional exhaustion, depersonalization, and low personal accomplishment. Later on, burnout was further described as a psychological syndrome [5]. The consideration of burnout as a syndrome has revolutionized its understanding. In the ICD-11 version, which has been valid since January 2022, burn-out is defined as a syndrome due to stress at work that cannot successfully be dealt with. The burnout syndrome has thus officially attained the status of an illness in connection to all occupational stress factors. It is explicitly stated that the syndrome should not be used to classify experiences in other areas of life, but should be limited to the workplace [6].

Burnout is an important phenomenon in the health care sector, especially among surgeons [7,8,9,10,11,12,13]. Accordingly, it is important to evaluate significant factors in the development of a burnout. In surgical disciplines, burnout seems to be more noticeable due to long working hours, delayed gratification, challenges in work–life balance and high standards related to patient care, especially in a changing healthcare environment [14,15].

In an international comparison, burnout rates of up to 19.6% are reported in surgical disciplines [16]. Studies showed that factors such as age, family status, specialty, number of night shifts per week, weekly work hours and remuneration independently influence burnout. Burnout is often accompanied by a lacking sense of professional fulfillment. Simultaneously, professional fulfillment seems to be the only predictor of satisfaction and the only sign of mental health in surgeons [16]. Hallsten [17] described a fragile self-concept, dependence on successful performance in a certain role and the will to achieve goals independently as key factors for burnout vulnerability. Fischer [18] as well as Hallsten [17] argued that narcissistic aspects were important factors in the development of a burnout crisis. Therefore, the association between narcissism and burnout is not new.

### 1.2. Narcissism

When considering narcissistic traits, subclinical narcissism has to be distinguished from its pathological form, the narcissistic personality disorder. However, the transition cannot be easily determined [19]. Narcissistic personality aspects have been described in form of a continuum [20,21,22]. Every personality contains narcissistic manifestations. Individuals can be distinguished by their respective scores. Accordingly, there is a threshold at which the level of manifestation is perceived as pathological. To define a cut-off value appears to be very difficult and must be tailored to the specific diagnostic procedure. However, this allows us to describe the trait’s nature by pathological characteristics which we can derive from the DSM-5. The manual describes nine diagnostic criteria: a grandiose sense of self-importance, being preoccupied with fantasies of unlimited success, power, brilliance, beauty, ideal love, a belief in the own uniqueness and entitlement, requirement of excessive admiration, the lack of empathy, as well as arrogant behaviors [23].

Specifically, narcissism and its pathological form, narcissistic personality disorder, express themselves through many different signs [24]. It is based on a strong insecurity about oneself, which is accompanied by an increased ability to offend. Many narcissists tend to cover those insecurities with exaggeration and self-promotion. A lack of empathy, egoism, overestimation of one’s own worth or an excessive attitude of entitlement can occur [25,26]. In pathological narcissism, often only the outwardly presented self-confidence appears to be present. Instead, affected people fluctuate between an exaggerated sense of grandeur and extreme feelings or fears of being small and insignificant. The environment often notices little of these fears because they are covered up with a grandiose self-dramatization [27].

### 1.3. Dimensions of Narcissism

Wink [28] wrote about the possible existence of two different subtypes of narcissism. Through statistical examination of self-report scales, he found a pattern suggesting a two-faced structure of narcissistic styles. On the one hand the vulnerability-sensitivity type, which is related to introversion, defensiveness, anxiety, and vulnerability, and on the other hand the grandiosity-exhibitionistic style, marked by extraversion, self-assurance, exhibitionism, and aggression. Ahn, Kwolek & Bowman [29] followed this concept in their study and were able to show interesting results. They suggested a different approach towards narcissism breaking the unidimensional concept down into two orthogonal constructs. Krizan & Johar [30], followed a similar theory outlining the two faces of narcissism. Nevertheless, they only distinguished between vulnerable and grandiose narcissism. In 2013, Back et al. [31] released a comprehensive paper showing results of a set of related empirical examinations regarding grandiose narcissism. Their constituting model, the narcissistic admiration and rivalry concept (NARC), further splits the grandiose style into two subtypes and claims to address cognitive, affective-motivational, as well as behavioral aspects. Furthermore, it explains the development of two different paths of maintaining a grandiose self from their intraindividual spring to their distinct social interaction outcomes. According to the concept, narcissistic admiration incorporates the facets striving for uniqueness, grandiose fantasies, and charmingness. This assertive self-enhancement strategy should lead to social potency through social interaction processes. On the contrary, there is a form of antagonistic self-protection, which encompasses devaluation of others, striving for supremacy, and aggressiveness leading to social conflicts. This dimension is called narcissistic rivalry. According to Miller et al. [32] dissent around narcissism occurs due to unclear distinction between its grandiose and vulnerable forms. In this respect, it seems promising to distinguish between subtypes regarding the prediction of burnout.

### 1.4. Narcissism and Burnout in Surgeons

Narcissism is a trait associated with surgeons in particular. Although surgeons exhibit a higher degree of narcissism than their medical colleagues [33], it is not higher than the general population [34]. However, the relationship between narcissism and burnout in surgeons has not yet been described. Martinez, Aytes & Escoda [35] examined the association of burnout with personality disturbances in dentists, finding that narcissistic and borderline personalities were the types of personalities most frequently found in individuals with a burnout syndrome. In their study, personality was assessed using the International Psychologic Disturbance Exam (IPDE). Thereby, narcissism was recorded as a personality disorder, not in terms of subclinical narcissism, like in the present study.

The relationship between narcissism and burnout has been studied in other populations revealing contradictions. Barnett & Flores [36] examined this issue regarding burnout in schools and found a positive association to narcissism. To measure narcissism, the Pathological Narcissism Inventory (PNI) and the overall pathological narcissism score were used. It did not distinguish between the different dimensions of narcissism. Schwarzkopf et al. [37] conducted a study among 723 in-house patients with job-stress related disorders. They found the general conception of overall narcissism to be significantly related to overall burnout. A dimension covering egocentrism and overestimation was positively correlated to emotional exhaustion and depersonalization. A subscale addressing the tendency to cope with emotional threats by identifying with ideal models showed a negative association with personal accomplishment. They concluded that narcissism plays an important role in the development of burnout. Studies by Prusik & Szulawski [38] and Känel et al. [39] deal with narcissism in work-related burnout. Prusik & Szulawski [38] examined the Relationship Between the Dark Triad Personality Traits, Motivation at Work, and Burnout Among HR Recruitment Workers. They found that employees with high levels of narcissism in HR environment are highly motivated and resilient towards burnout. In this study, narcissism was assessed using the Short Dark Triad questionnaire. Although subclinical narcissism was recorded, it was described as an overall construct. Table 1 summarizes the results of previous studies on this topic.

Except for Känel et al. [39], none of the previous studies distinguished between the dimensions of narcissism. Känel et al. [39] also investigated the interrelation in a working population. They differentiate between two forms of narcissism, adaptive and maladaptive narcissism, yielding interesting results. In doing so, they define adaptive narcissism as the talent to influence people, seeing oneself as a good leader, the willingness to make decisions, feeling insecure about actions and recognition of one’s authority. Maladaptive narcissism is captured, as easy for participants to manipulate people, depending on whether they get the respect that they feel entitled to, if they expect a lot from other people and never feeling satisfied until getting what they feel they deserve. Maladaptive narcissism led to higher levels of burnout, while adaptive narcissism was associated with lower levels of burnout. However, only two scales of the NPI were used for the distinction. Nevertheless, the results indicate that the relationship between the constructs narcissism and burnout is more differentiated when narcissism is broken down into subtypes.

Consequently, the aim of this study was to evaluate whether a differentiated view on the construct of narcissism can resolve formerly described contradictory results. In the present study, we focus on the dimensions of grandiose narcissism, namely admiration and rivalry. Studies previously following this subtype approach found that admiration tends to be associated with positive outcomes, whereas rivalry seems to be associated with negative outcomes [31]. An explanation for this could be that admiration is highly positively correlated with self-esteem, extraversion, and openness, among others, whereas rivalry positively correlates with neuroticism and negatively correlates with self-esteem, agreeableness, and conscientiousness.

### 1.5. Aim of this Study and Hypotheses

Our aim was to examine how grandiose narcissism and burnout are related. Up to now, no results were derived out of the chosen instruments. However, previous study results suggest that narcissistic admiration will predict negative correlations with emotional exhaustion and depersonalisation but positive correlations with personal fulfilment, whereas rivalry will predict positive correlations with emotional exhaustion and depersonalisation but negative correlations with personal fulfilment [31]. For this reason, we derived the following hypotheses.

**Hypothesis 1.**  *Admiration is negatively related to emotional exhaustion.*

**Hypothesis 2.**  *Admiration is positively related to personal fulfilment.*

**Hypothesis 3.**  *Admiration is negatively related to depersonalisation.*

**Hypothesis 4.**  *Rivalry is positively related to emotional exhaustion.*

**Hypothesis 5.**  *Rivalry is negatively related to personal fulfilment.*

**Hypothesis 6.**  *Rivalry is positively related to depersonalisation.*

## 2. Materials and Methods

### 2.1. Participants

This study was approved by our institution’s ethics review board (EKEFH01/22). The sample encompasses surgeons from all over Germany, working in clinics and hospitals as prior research suggested these are associated with high levels of narcissism [33].We further chose this clientele because it offered a homogenous sample, which would fit our choice of instruments, in particular regarding the MBI, which was developed for health-care personnel.

The subjects were questioned via email, requesting them to complete a standardized online-survey containing the two inventories, MBI and NARQ, as well as some demographics. To generate the sample, the website www.deutsches-krankenhaus-verzeichnis.de (accessed on 25 September 2022) was used. All surgeons listed on the homepages of surgical clinics were solicited. A total of 11,400 emails were initially sent. 1427 participants took part in the study of whom 1390 physicians completed the questionnaires in full. No compensation was offered or recieved for participating in the study.

A priori power analyse was run with G*Power version 3.1.9 to determine the optimal sample size. For a *f*^2^ = 0.05, α error probability = 0.001 and desired power (1 − β error probability) = 0.80 and 2 predictors a total sample size of 314 is necessary.

We were able to analyze data from 352 participants. Cases where data was incomplete were excluded. The sample population contained 113 female physicians (32.1%). The age ranged from 25 to 66 years (*M* = 42.6; *SD* = 14.0). Two-hundred-eighty-two (80.1%) individuals were married or lived in a permanent relationship. The average number of children was 1.4 (*SD* = 1.3). The individuals reported an average workload of 57.1 h per week (*SD* = 11.7). Work experience in their job was reported from zero to 40 years (*M* = 15.0; *SD =* 10.6).

### 2.2. Measures

Burnout was measured by the German version of the Maslach Burnout Inventory (MBI-d) by Büssing and Perrar [40]. It consists of three scales assessing the dimensions depersonalization; *DP* with five items, emotional exhaustion; *EE* with nine items, and personal accomplishment; *PA* with eight items. Participants can rate frequencies in a six-point likert scale, ranging from “1 = never” over “2 = very rarely”, “3 = rather rarely”, “4 = sometimes”, “5 = rather often” to “6 = very often”. Scores were added up for each scale. For data analysis, low personal accomplishment was reversed because of its inverse item-formulations. From here, this subscale is called personal fulfillment.

In our sample, Cronbach’s alpha was 0.89 for emotional exhaustion, 0.71 for depersonalization, 0.77 for personal accomplishment, and 0.88 for the MBI total scale, indicating acceptable to good internal consistency.

Narcissism was measured by the Narcissistic Admiration and Rivalry Questionnaire (NARQ), developed by Back et al. [31]. It is available in German and is composed out of a Likert-type for the two scales narcissistic admiration and narcissistic rivalry. Each consists of three facets with three items. In total, the inventory encompasses 18 items where subjects can agree or disagree from “1 = not agree at all” to “6 = agree completely”. The facets are mentioned and explained above. It is possible to calculate scores for every level of the NARQ beginning from facets up to an overall score for grandiose narcissism. Cronbach’s Alpha for the two scales are 0.87 (admiration) and 0.83 (rivalry).

### 2.3. Statistical Analysis

To analyse the collected data, we used Jamovi 2.3.15 [41]. Descriptive statistical analyses were run to characterize data and our sample [42]. Outliers were analysed with box plots which tested with studentized residuals (±3) and would be excluded from the analysis. Normal distribution was assessed with Shapiro–Wilk’s test (*p* > 0.05). Pearsons product-moment-correlation was run to assess relationships between emotional exhaustion, personal fulfilment, depersonalisation, NARQ admiration and NARQ rivalry [43].

Stepwise linear Regression was run to understand the effect of narcissism dimensions measured with NARQ on our dependent variables. Two models for each MBI scale were generated. AIC, BIC (focusing on the least values) and a overall model F-Test was used to determine the most relevant model. Model 1 was set up with the demographic variables sex, marital status, age and workload. Model 2 was set up with narcissism dimensions The two constructs are different in their stability over time. While grandiose narcissism is thought as a classical trait of stability over time, there has to be a source of cognitive and behavioral dynamic that influences burnout [21,31]. Burnout has to be regarded as a state, because it is not a set of stable attributes. It rather is a risk state which might lead to pathologic disorders [40,44]. This understanding influences our statistical modeling. Therefore, narcissism must serve as the independent, and burnout as the dependent variable.

Linearity was assessed visual with scatterplots and validated with results from correlation analyses. Homoscedasticity was assessed with Q-Q plots and normality of the residuals were assessed with residual plots from the jamovi regression package. Autocorrelation was assessed with Durbin-Watson-Test and collinearity was assessed with Variance Inflation Factor (VIF). VIF exceeding 2.5 would indicate collinearity [45].

## 3. Results

### 3.1. Descriptive Statistics and Correlations between Variables

Means and standard deviations for the subscales of all three inventories are depicted in Table 2. Table 3 shows the results of the correlation analysis.

### 3.2. Requirements

To assess linearity scatterplots of dependent variables personal fulfilment, depersonalisation, and emotional exhaustion against independent variables with superimposed regression lines were plotted. Visual inspection of these plots indicated a linear relationship between the variables. In all analyses, there was homoscedasticity and normality of residuals, assessed with Q-Q-Plots. Collinearity statistics and Durbin-Watson-Test for autocorrelation met the requirement criteria. Outliers were identified with boxplots and were removed from respective analyses as they were not representing the target population.

The assumption of normality for NARQ rivalry values was not met. Because the distribution was left-skewed, all values were logarithmized, achieving an assumption of normal distribution.

### 3.3. Emotional Exhaustion

A linear regression was run to understand the effect of narcissism dimensions on scores of emotional exhaustion. 34 participants were outliers and were removed from the analyses. Model 1 consisting of demographic variables gender, age, marital status, workload and month in job was significant (*p* = 0.01) and model 2 was significantly different from model 1 with an improvement of R^2^ by 4.65% (F(2, 310) = 7.96, *p* < 0.001) (Table 4). The prediction equation was: Emotional Exhaustion = 21.80 − 2.45 × Gender − 0.16 × NARQ-Admiration + 0.45 × NARQ-Rivalry (Table 5). This model statistically significantly predicted personal fulfillment, F(7, 310) = 4.58, *p* < 0.001, accounting for 9,37% of the variation in emotional exhaustion with adjusted R^2^ = 7.33%, representing a small size effect according to Cohen [46] (Table 6).

### 3.4. Personal Fulfillment

A linear regression was run to understand the effect of narcissism dimensions on scores of personal fulfillment. Forty-four participants were outliers and were removed from the analyses. Model 1 consisting of demographic variables gender, age, marital status, workload and month in job was significant (*p* < 0.001) and model 2 was significantly different from model 1 with an improvement of R^2^ by 12.1% (F(2, 300) = 22.7, *p* < 0.001) (Table 7). The prediction equation was: personal fulfillment = −18.55 + 0.09 × Gender + 0.18 × NARQ-Admiration − 2.49 × NARQ-Rivalry (Table 8). This model statistically significantly predicted personal fulfillment, F(7, 300) = 10.64, *p* < 0.001, accounting for 19.89% of the variation in emotional exhaustion with adjusted R^2^ = 18.02%, representing a medium size effect according to Cohen [46] (Table 9).

### 3.5. Depersonalisation

A linear regression was run to understand the effect of narcissism dimensions on scores for depersonalisation. Thirty-two participants were outliers and were removed from the analyses. Model 1 consisting of demographic variables gender, age, marital status, workload and month in job was significant (*p* < 0.01) and model 2 was significantly different from model 1 with an improvement of R^2^ by 4.66% (F(2, 309) = 7.94, *p* < 0.001) (Table 10). The prediction equation was: depersonalisation = 6.88 × NARQ-Rivalry (Table 11). This model statistically significantly predicted personal fulfillment, F(7, 309) = 4.57, *p* < 0.001, accounting for 9.38% of the variation in emotional exhaustion with adjusted R^2^ = 7.83%, representing a medium size effect according to Cohen [46] (Table 12).

Additionally, our results can be summarized by the central correlation and regression coefficients to characterize the differential relationships between burnout indicating scales and narcissism scales (Table 13).

## 4. Discussion

Campbell [47] asks the legitimate question: Is narcissism really so bad? However, this is not an easy question to be answered. Campbell [47] argues that it is appropriate not to classify narcissism as a negative personality trait per se. He even hypothesizes that narcissism may be a functional and healthy strategy for dealing with the modern world, as research indicates that narcissists are not necessarily fragile, depleted, or depressed.

The results of our study indicate that a differentiated view on the construct of narcissism allows to equally highlight the positive aspects and the dark sides of narcissism. The purpose of the present study was to investigate the relationship of the constructs of narcissism and burnout. It was proposed that the dimensions of grandiose narcissism would predict burnout severity.

Back et al. [31] (2013) developed the NARC process model, which identifies two positively correlated but distinct dimensions: rivalry and admiration. Here, it is assumed that the overarching goal of maintaining the grandiose self can be pursued through two social strategies. Assertive self-enhancement is manifested by the tendency to seek social admiration through self-presentation, whereas antagonistic self-protection represents the tendency to prevent social failure through self-defense. Both strategies are conceptualized on the affective-motivational, cognitive, and behavioral levels. The NARC concept by Back et al. [31] postulates that admiration leads to positive intrapersonal and interpersonal adjustment indicators. Additionally, there is a higher emotional stability, extraversion and openness to new experiences and positive behaviors, which have long-term, positive effects on life success. These include increased involvement in social interactions and pro-social behavior. This dynamic, though perceived positive effect, might serve as a resilience-promoting factor against external stressors. In contrast, narcissistic rivalry rather leads to negative intrapersonal and interpersonal adjustment indicators. In addition, there is lower emotional stability, agreeableness, conscientiousness, and higher scores in neuroticism, impulsivity, and anger. The resulting social conflict poses a threat to the individual’s self-concept. In relation to our findings, it appears that surgeons with high levels of admiration find their profession fulfilling and are less emotionally exhausted. Personal fulfilment may improve surgeons’ interpersonal effectiveness and non-technical skills (such as teamwork, communication, and cooperation). Consequently, the surgical performance [48] of the individual and the entire team might improve [49,50].

Moreover, surgeons with high levels of rivalry show the opposite effect. These surgeons are more emotionally exhausted and depersonalised experiencing their profession as less fulfilling. In clinical practice, this indicates that disruptive behaviours are detrimental to patients [51]. This seems to be associated with increased medical errors, reduced well-being, high turnover and impairment of collaboration in the perioperative environment [52,53,54].

Our findings indicate that narcissistic admiration appears to be a protective personality trait, while rivalry appears to increase the severity of burnout. However, it has to be considered that admiration is not inevitably a desirable trait. Prusik & Szulawski [38] and Miller, Lynam, Hyatt & Campbell [32] correctly point out that individuals with a high level of admiration may be resistant to burnout, but may still exhibit undesirable features.

Maslach et al. [4] showed that individuals who score higher on neuroticism and lower on self-esteem are more prone to burnout. This is supported by recent literature, e.g., a meta-analysis by Alarcon, Eschleman and Bowling [55]. Back et al. [31] also reported that admiration is positively associated with self-esteem and negatively associated with neuroticism. The opposite is true for rivalry. Therefore, self-esteem should be regarded as a decisive mediator. Evidence for this assumption is also provided by Rosse, Boss, Johnson & Crown [56], Neumann [20] and Schwarzkopf et al. [37]. Other possible mediators or moderators such as adaptive coping strategies and work-level stress should be considered to explain the relationships found in this study as well.

Results presented in Table 1 finding narcissism to promote burnout, can be interpreted in a differently. Schwarzkopf et al. [37], for example, concluded that narcissism plays an important role in the development of burnout. However, when interpreting the results, it should be considered that especially patients who were treated for burnout did not have effective adaptive strategies to cope with work-related stress. This could particularly include participants showing high levels of rivalry. Our findings suggest that individuals with higher admiration are less likely to suffer from burnout and therefore are less likely to undergo therapy. This highlights the importance of looking at narcissism from various perspectives when interpreting results of other studies.

It would therefore also be of interest to investigate whether the results of Barnett & Flores [36], who found that narcissism was positively associated with school burnout, can be replicated if the dimensions of narcissism are used rather than the overall construct. Prusik & Szulawski [38], who draw the opposing conclusion from their findings, state that employees with high levels of narcissism in a HR environment are highly motivated and resilient to burnout. Again, in their study subclinical narcissism was surveyed as an overall construct. Considering the current research, it is reasonable to assume that this applies primarily to individuals with high levels of admiration and less to individuals with high levels of rivalry. In view of the items used in the questionnaire, it becomes clear that they refer primarily to facets of admiration and hardly capture rivalry. Therefore, the results might differ if the two forms of grandiose narcissism were explicitly measured. In this context, Rogoza, Wyszyńska, Maćkiewicz, & Cieciuch [57] state that the NARC model may be a promising approach to resolve inconsistencies in current research literature.

## 5. Conclusions

Rogoza, Żemojtel-Piotrowska, Kwiatkowska & Kwiatkowska, K. [58] examined the blue face of narcissism in addition to the bright and dark face of narcissism examined in this study. We focused only on the dimensions of grandiose narcissism due to our specific sample population containing only surgeons. In future studies, it would be interesting to include vulnerable narcissism in the analysis to uncover the effects on burnout compared to admiration and rivalry. This would address Miller, Lynam, Hyatt & Campbell’s [32] call to develop a unified construct. In their view the controversies surrounding narcissism result from unclear distinctions between its grandiose and vulnerable forms.

In this respect, the goal of future research should be to precisely capture narcissism in all its facets in order to obtain comparable results and resolve controversies about narcissism.

## Figures and Tables

**Table 1:** Overview of studies on the relationship between narcissism and burnout.

Author	Title	Study Purpose	Moderator	Sample	Questionnaires	Results
Barnett & Flores (2016) [36]	Narcissus, exhausted: Self-compassion mediates the relationship between narcissism and school burnout.	Relationships between narcissism, self-compassion, and school burnout	Self-compassion	813 undergraduate students	Pathological Narcissism Inventory (PNI) School Burnout Inventory (SBI) Self-Compassion Scale (SCS)	Narcissism was positively associated with school burnout and had an indirect effect on school burnout through self-compassion, specifically through the over-identified and isolation components of self-compassion.
Schwarzkopf et al. (2016) [37]	Empirical evidence for a relationship between narcissistic personality traits and job burnout	Association between narcissism and job burnout adjusting for gender, age, socioeconomic status, depressive symptom severity, sleep quality, and perceived stress		723 clinic patients with job-stress related disorders	Maslach Burnout Inventory (MBI) Narcissism Inventory (NI-90)	After controlling for sociodemographic factors, depressive symptoms, sleep quality, and perceived stress, the magnitude of the relationship between narcissism and burnout was comparable to that between depressive symptom severity and burnout
Prusik & Szulawski (2019) [38]	The relationship between the dark triad personality traits, motivation at work, and burnout among HR recruitment workers	Relationship between the dark triad personality traits, burnout and motivational factors	Motivation	175 recruiters from internal and external HR departments	Dark Triad of Personality Test (D3-Short) Barbuto’s Motivation Sources Inventory Oldenburg Burnout Inventory (OLBI)	A higher level of narcissism was related to both internal and external motivations and was not associated with disengagement and exhaustion.
Känel et al. (2017) [39]	Association of adaptive and maladaptive narcissism with personal burnout: findings from a cross-sectional study	Association between narcissism and personal burnout adjusting for age, gender, depression, sleep quality, stress at work and at home		1461 employees of a pharmaceutical company	Narcisstic Personality Inventory (NPI) Copenhagen Burnout Inventory (CBI)	Higher adaptive narcissism was associated with lower burnout scores and higher maladaptive narcissism was associated with higher burnout scores.

**Table 2:** Descriptive Statistics.

Inventory	Variable	*M*	*SD*
MBI-d	Emotional Exhaustion	3.04	0.91
	Personal Fulfillment	2.17	0.50
	Depersonalization	2.61	0.85
NARQ	Narcissistic Admiration	2.76	0.86
	Narcissistic Rivalry	1.76	0.69

Note. *n* = 352. Scale Ranges MBI-d (1–6); NARQ (1–6).

**Table 3:** Correlation matrix of the relevant variables.

	Personal Fulfillment	Depersonalisation	Emotional Exhaustion	NARQ Admiration
Depersonalisation	−0.322 ***	—		
Emot. Exhaustion	−0.382 ***	0.559 ***	—	
NARQ Admiration	0.223 ***	0.167 **	0.044	—
NARQ Rivalry	−0.103	0.438 ***	0.253 ***	0.569 ***

Note. ** *p* < 0.01, *** *p* < 0.001.

**Table 4:** Model Comparison for Emotional Exhaustion.

Comparison						
Model	Model	ΔR^2^	F	df1	df2	*p*
1	2	0.05	7.96	2.00	310	<0.001

Note. R^2^_adj_ = adjusted R^2^.

**Table 5:** Linear Regression Analysis for Emotional Exhaustion.

			95% CI					95% CI	
Predictor	B	SE	Lower	Upper	t	*p*	β	Lower	Upper
Intercept	21.80	3.17	15.57	28.03	6.88	<0.001			
Gender:									
female–male	−2.45	0.98	−4.39	−0.52	−2.50	0.01	−0.31	−0.55	−0.06
Age	−0.02	0.03	−0.08	0.05	−0.59	0.56	−0.03	−0.15	0.08
Marital Status:									
partnership–single	−0.62	1.11	−2.81	1.57	−0.56	0.58	−0.08	−0.35	0.20
Workload	0.10	0.04	0.02	0.18	2.35	0.02	0.13	0.02	0.23
Moth in Job	0.02	0.05	−0.08	0.13	0.46	0.64	0.02	−0.08	0.13
NARQ Admiration	−0.16	0.07	−0.29	−0.03	−2.49	0.01	−0.15	−0.26	−0.03
NARQ Rivalry	0.45	0.10	0.24	0.66	4.29	< 0.001	0.26	0.14	0.37

Note. B = Regression Coefficient SE = Standard Error, t = t-statistics, *p* = significance, β = standardizised Regression Coefficient.

**Table 6:** Overall Model Test for Emotional Exhaustion.

				Overall Model Test			
Model	R	R^2^	R^2^_adj_	F	df1	df2	*p*
1	0.22	0.05	0.03	3.09	5	312	0.01
2	0.31	0.09	0.07	4.58	7	310	<0.001

Note. R^2^_adj_ = adjusted R^2^.

**Table 7:** Model Comparison for Personal Fullfillment.

Comparison						
Model	Model	ΔR^2^	F	df1	df2	*p*
1	2	0.12	22.70	2.00	300	<0.001

Note. R^2^_adj_ = adjusted R^2^, ΔR^2^ = delta R^2^ model 1 and R^2^ model 2.

**Table 8:** Linear Regression Analysis for Personal Fullfillment.

			95% CI					95% CI	
Predictor	B	SE	Lower	Upper	t	*p*	β	Lower	Upper
Intercept	−18.55	2.25	−22.98	−14.12	−8.25	<0.001			
Gender:	0.09	0.02	0.05	0.12	4.82	<0.001	0.29	0.17	0.41
female–male									
Age	−0.40	0.40	−1.18	0.39	−1.00	0.32	−0.13	−0.37	0.12
Marital Status:									
partnership–single	−0.12	0.44	−0.98	0.73	−0.28	0.78	−0.04	−0.31	0.23
Workload	0.01	0.05	−0.08	0.11	0.24	0.81	0.01	−0.09	0.12
Moth in Job	0.01	0.02	−0.03	0.04	0.29	0.77	0.02	−0.09	0.12
NARQ Admiration	0.18	0.03	0.13	0.23	6.73	<0.001	0.41	0.29	0.53
NARQ Rivalry	−2.49	0.70	−3.87	−1.12	−3.58	<0.001	−0.22	−0.34	−0.10

Note. B = Regression Coefficient SE = Standard Error, t = t-statistics, *p* = significance, β = standardizised Regression Coefficient.

**Table 9:** Overall Model Test for Personal Fullfillment.

			Overall Model Test			
Model	R	R^2^	F	df1	df2	*p*
1	0.28	0.08	5.11	5	302	<0.001
2	0.45	0.20	10.64	7	300	<0.001

Note. R^2^_adj_ = adjusted R^2^.

**Table 10:** Model Comparison for Depersonalisation.

Comparison						
Model	Model	ΔR^2^	F	df1	df2	*p*
1	2	0.05	7.94	2.00	309	<0.001

Note. R^2^_adj_ = adjusted R^2^, ΔR^2^ = delta R^2^ model 1 and R^2^ model 2.

**Table 11:** Linear Regression Analysis for Depersonalisation.

			95% CI					95% CI	
Predictor	B	SE	Lower	Upper	t	*p*	β	Lower	Upper
Intercept	10.13	5.727	−1.14	21.4	1.769	0.078			
Gender:	−0.048	0.047	−0.141	0.044	−1.025	0.306	−0.065	−0.189	0.060
female–male									
Age	−1.716	1.034	−3.75	0.318	−1.66	0.098	−0.218	−0.477	0.041
Marital Status:									
partnership–single	−0.869	1.131	−3.094	1.357	−0.768	0.443	−0.111	−0.394	0.173
Workload	0.127	0.125	−0.120	0.373	1.011	0.313	0.057	−0.054	0.167
Moth in Job	0.110	0.049	0.014	0.206	2.262	0.024	0.125	0.016	0.233
NARQ Admiration	−0.160	0.070	−0.300	−0.030	−2.380	0.020	−0.150	−0.270	−0.030
NARQ Rivalry	6.88	1.74	3.45	10.31	3.94	<0.001	0.250	0.130	0.380

Note. B = Regression Coefficient SE = Standard Error, t = t-statistics, *p* = significance, β = standardizised Regression Coefficient.

**Table 12:** Overall Model Test for Emotional Exhaustion.

				Overall Model Test			
Model	R	R^2^	R^2^_adj_	F	df1	df2	*p*
1	0.22	0.05	0.03	3.08	5	311	0.01
2	0.31	0.09	0.07	4.57	7	309	<0.001

Note. R^2^_adj_ = adjusted R^2^.

**Table 13:** Summarizing Table.

	Emotional Exhaustion				Personal Fullfillment				Depersonalisation			
Predictor	*r*		β		*r*		β		*r*		β	
NARQ Admiration	0.04		−0.16	**	0.22	***	0.18	***	0.17	**	−0.16	*
NARQ Rivalry	0.25	***	0.45	***	−0.10		−2.49	***	0.44	***	6.88	***

*r* = correlation coefficient, β = standardized regression coefficient. *p* < 0.05: *, *p* < 0.01: **, *p* < 0.001: ***.

## Data Availability

The datasets used and/or analysed during the study are available from the corresponding author on reasonable request.
